# N-acetylcysteine stimulates organelle malfunction in endometriotic cells via IFN-gamma signaling

**DOI:** 10.1038/s41598-025-00195-z

**Published:** 2025-04-29

**Authors:** Elif Karakoç, Sevil Oskay Halaçlı, Rumeysa Havvanur Hanelçi, Selda Ayhan, Cemil Can Eylem, Emirhan Nemutlu, Pergin Atilla

**Affiliations:** 1 Faculty of Medicine, Department of Histology and Embryology, Hacettepe University, Ankara, 06230 Turkey; 2 Division of Pediatric Immunology, Department of Basic Sciences of Pediatrics, Hacettepe University, Institute of Childs’ Health, Ankara, 06230 Turkey; 3 Translational Medicine Laboratories, Faculty of Medicine, Hacettepe University, Ankara, Turkey; 4 Division of Pediatric Metabolism, Department of Basic Sciences of Pediatrics, Institute of Childs’ Health, Hacettepe University, Hacettepe University Faculty of Medicine, Ankara, 06230 Turkey; 5 Faculty of Pharmacy, Department of Analytical Chemistry, Hacettepe University, Ankara, 06230 Turkey

**Keywords:** Endometriosis, N-acetyl cysteine, IFN-ɣ, ER stress, Mitochondrial dysfunction, Metabolomics, Cell biology, Organelles, Immunopathogenesis, Inflammation, Infertility, Urogenital reproductive disorders

## Abstract

Endometriosis is a chronic inflammatory gynecologic disease characterized by the abnormal implantation of endometrial tissue outside the uterus. The inflammatory microenvironment of endometriosis is dominated by highly migratory endometriotic cells, inflammatory cells, and cytokines. There is no curative treatment other than oral contraceptives, painkillers, and surgery. N-acetyl-L-cysteine (NAC), an anti-inflammatory compound has been identified as a promising agent for endometriosis. However, it is still unclear how NAC interacts with interferon-gamma (IFN-ɣ) and common cytokines in the endometriotic microenvironment. This study aimed to investigate the effects of NAC, alone and in combination with IFN-ɣ and major cytokines such as interleukin-6 (IL-6), tumor necrosis factor-alpha (TNF-⍺) on endometriotic cells. For this purpose, we performed a real time-dependent cell impedance assay, Annexin V/PI and ER tracking by flow cytometry, immunofluorescence, western blotting, and metabolomic assays. Our results offered a new insight into the complex relationship between NAC and IFN-ɣ, both of which reduced endometriotic cells’ proliferation, induced ER stress and mitochondrial dysfunction. In conclusion, NAC and IFN-ɣ, alter the metabolism of endometriotic cells, leading to endoplasmic reticulum stress and mitochondrial dysfunction. These findings suggest that NAC when combined with IFN-ɣ, has the potential to generate innovative therapeutic modalities for the treatment of endometriosis.

**Supplementary Information:**

The online version contains supplementary material available at 10.1038/s41598-025-00195-z.

## Introduction

Endometriosis, an enduring inflammatory gynecologic disorder, is characterized by the aberrant placement of endometrial glands and stromal tissue outside the uterine cavity^1–4^. The condition is expected to impact 10% of reproductive-age women, and 190 million women estimated to be affected globally^1,2,5,6^. The most common symptoms include chronic pelvic pain, dysmenorrhea, and dyspareunia, with the condition often leading to infertility^2,5,7,8^. Although the pathophysiology of endometriosis is not fully explained, retrograde menstruation is the most common theory, and endometriotic cells are known to have a high migratory capacity, be resistant to apoptosis and induce inflammation^3,5,8–11^. Endometriosis results in chronic inflammatory dysregulation, which raises the risk of ovarian cancer as well as immunological and metabolic issues^2^. Current treatments, including laparoscopic excision, hormonal therapy and non-steroidal anti-inflammatory drugs, provide relief but are not curative^1,5,7,12^.

Innate immune cells, such as natural killer cells (NKs), neutrophils, and macrophages, contribute to the inflammatory microenvironment of endometriosis by secreting cytokines, including interleukin-1 (IL-1), interleukin-6 (IL-6), tumor necrosis factor alpha (TNF-⍺), and interferon gamma (IFN- ɣ)^8,9,13^. While TNF-⍺ and IL-6 are well-detailed promoters of endometriotic proliferation and correlate disease severity, the role of IFN-ɣ signaling remains unclear^14,15^. IFN-ɣ, inhibits apoptosis and stimulates the adhesion of ectopic endometrial cells to the peritoneum^10^. Although some studies report elevated IFN-ɣ levels in the peritoneal fluid of endometriosis patients, others suggest significantly lower IFN-ɣ levels^16,17^. These conflicting findings highlight the need for further research to precise IFN-ɣ’s role in endometriosis progression.

N-acetylcysteine (NAC) is an antioxidant compound known for its anti-inflammatory and antiproliferative effects on endometriotic cells and tissue^18^. Recent studies have shown that NAC alters the expression of proliferative, differentiation-related, and inflammatory proteins and genes in endometriotic cells. Clinically, NAC treatment significantly reduced endometriotic cyst volume and symptoms in women with ovarian endometriosis^19^. Anastasi et al. demonstrated that NAC not only reduced the size of endometriomas, and serum CA125 levels but also reduced pain symptoms and improved fertility outcomes in affected individuals^19^. In our previous study, we detected that NAC inhibited the migratory capacity of endometriotic cells, suppressed cell proliferation, and induced ER stress, suggesting a potential mechanism through which NAC exerts its therapeutic effects^20^.

Given the conflicting evidence regarding the role of IFN-ɣ in endometriosis and the emerging significance of NAC in modulating endometriotic cell function, this study aims to elucidate whether NAC influences IFN-ɣ -mediated inflammatory signaling and cellular metabolism of endometriotic cells.

We investigated the effects of NAC both alone and in combination with IFN-ɣ and other cytokines, on endometriotic cell responses with a focus on ER stress induction, metabolic pathway alterations, and proliferation inhibition.

Our findings indicate that NAC, either independently or in presence of IFN-ɣ, induces ER stress, disrupts metabolic pathways, and inhibits endometriotic cell proliferation, while IFN-ɣ receptor blockade modifies these effects. This study provides novel mechanistic insights and highlights the potential therapeutic implications of NAC in the management of endometriosis.

## Materials and methods

### Study design

A controlled in vitro study was designed to investigate the effects of NAC and cytokines on endometriotic and endometrial stromal cells. The human endometriotic stromal cell line (12Z) and human stromal endometrial cell line (HESC) and were used. The experimental outcomes were evaluated using xCELLigence, flow cytometry, immunofluorescence, western blotting, and metabolomics. Biological replicates were calculated using power analysis (G-Power v3.1).

### Cell culture

The human endometrial stromal cell line (HESC) (#T0533, ABM, Canada) was cultured in DMEM/F12 medium (#P04-41550, PAN-Biotech, USA) supplemented with 10% fetal bovine serum (FBS) and 1% penicillin-streptomycin. The human endometriotic stromal cell line (12Z) (#T0764, ABM, Canada) was cultured in Prigrow IV medium (#TM004, ABM, Canada) supplemented with 10% FBS. Cells were maintained at 37 °C in an incubator with 5% CO_2_. Mycoplasma contamination was tested before experiments, and cells were subcultured for assays upon reaching 80% confluence^20^.

### Real-time cell impedance assay

When the 12Z and HESC cell lines reached 70–80% confluence, the cell culture medium was removed, and the cells were washed with PBS. Next, the cells were treated with a trypsin/EDTA solution (#T4049, Sigma-Aldrich, USA) for 10 minutes at 37°C and then centrifuged at 1200 rpm. Finally, 5000 cells/200 µL per well were seeded into the 96 wells of the gold electrode-covered ‘‘E plates’’. The impedance of cell pressure on the base was continuously recorded to create the ‘cell index’ using xCELLigence (RTCA, Agilent Technologies, USA). The IC50 dose of NAC (#A9165, Sigma-Aldrich, USA), which we had determined in our previous study (3.87 × 10^− 9^ M) (*n* = 5)^20^. We also applied individually or NAC-combined doses of TNF-⍺ (#SRP3177, Sigma-Aldrich, USA), (100 µM); IL-6 (#130-093-929, Miltenyi Biotec, Germany) (100 ng/mL); and IFN-ɣ (#285-IF-100/CF, R&D, USA) (100 ng/mL) (*n* = 5). We also evaluated the impact of NAC and IFN-ɣ on cell proliferation in presence of IFN-ɣ receptor antagonist (#HY-P4717, MedChem, USA) (35 µM/mL) (*n* = 5) on 12Z cells. The control group for each cell was treated only with cell media (*n* = 5). Cellular indices were monitored every 15 min for a total of 96 h^20^.

### Flow cytometric analyses

#### Apoptosis assay

HESC and 12Z cell lines were incubated with NAC, IL-6, TNF-⍺, and IFN-ɣ at the doses described in the xCELLigence assay. After treatment, cells were labeled with Annexin V/Propidium Iodide (#640914, Biolegend, USA) for 15 min in the dark (*n* = 3). The samples were then analyzed by flow cytometry using a Becton Dickinson (San Jose, CA, USA) flow cytometer and a Beckman Coulter Cytoflex Cytometer Software (v 1.2.5.3891).

#### ER tracking assay

HESC and 12Z cell lines were incubated with the compounds and labeled with and ER-Tracker Green (#E34251, Invitrogen, USA) (1 µM) (*n* = 3) in the dark for 30 min and analyzed by flow cytometry using a Becton Dickinson (San Jose, CA, USA) flow cytometer and a Beckman Coulter Cytoflex Cytometer Software (v 1.2.5.3891).

### Immunofluorescence labeling

The 12Z cells were expanded in 8-well chambered slides and treated with combinations or individual applications of NAC, IFN-ɣ, and IFN-ɣ receptor antagonist (Anta) (*n* = 3). After removing the culture media, the cells were washed with phosphate-buffered saline (PBS) (3 × 5 min) and fixed with 4% paraformaldehyde (#P6148, Sigma-Aldrich, Germany) for 10 min at room temperature (RT). Permeabilization was performed using Triton X-100 (#9036-19-5, Sigma-Aldrich, Germany) for 10 min RT. To prevent non-specific binding, cells were blocked with 5% bovine serum albumin (#A5611, Sigma-Aldrich, Germany) for 1 h at RT. Primary antibodies, anti-Ki 67 (#sc23900, Santa Cruz Biotechnology, USA) and anti-phospho IRE1 alpha (p-IRE1) (#AF7150, Affinity Biosciences, USA) were applied at a 1:100 dilution for 3 h at RT. Secondary antibodies, Alexa 596 (#A-11012, Thermo Scientific, USA) and Alexa 488 (#A-21202, Thermo Scientific, USA) were used at 1:1000 dilution for double labeling for 30 min at RT. Nuclei were counterstained with DAPI (#422801, Biolegend, USA). After mounting with antifade mounting media, slides were examined under a microscope, attached to a digital camera (Leica DMB6B, DFC7000T, LASV3 Wetzlar, Germany). A minimum of 5 non-overlapping images were captured per wells and ImageJ was used to calculate corrected total cell fluorescence (CTCF): CTCF = Integrated Density – (Area of selected cell x Mean fluorescence of background readings)^21^.

### Western blotting

The cells were washed twice with PBS and lysed using radioimmunoprecipitation (RIPA) Lysis Buffer System (#sc-24948, Santa Cruz Biotechnology, USA). The protein concentration of the resulting lysates was determined using the Quick Start Bradford Protein Assay Kit 2 (#5000202, Bio-Rad, USA). Proteins (20 µg) were electrophoresed on 10% SDS-PAGE gels (TGX FastCast 10%, Acrylamide Kit; #1610173, Bio-Rad, USA). The protein was transferred onto a prewet polyvinylidene difluoride membrane (100% methanol, 2 min) (Trans-Blot Turbo RTA Mini PVDF Transfer Kit; #1704272 and Trans-Blot Turbo Transfer System; #1704150; Bio-Rad, USA). The membrane was rinsed with Tris-buffered saline with Tween 20 (TBST), and nonspecific binding was blocked with 5% nonfat dry milk dissolved in TBST. The membrane was then incubated with the anti-IRE1 alpha (Inositol requiring enzyme-1 alpha) (p Ser724) antibody (dilution 1:1000; #NB100-2323, Novus, USA) and β-actin (1:5000; #MA5-15739, Invitrogen, USA) primary antibody overnight at 4 °C, washed with TBST, incubated with HRP-conjugated secondary antibody (Anti-rabbit IgG, HRP-linked Antibody; #7074, Sigma-Aldrich, Germany) cell signaling for IRE1, and HRP Goat Anti-Mouse IgG (H + L) (#AS003, ABclonal, USA) for actin (1 h; room temperature; dilution, 1:5,000). Washed with TBST, and detected with the Clarity Western ECL Substrate (#1705061, Bio-Rad, USA) by the SYNGENE G: Box Chemi XRQ. Quantification was performed using ImageJ.

### Metabolomics analysis

GC–MS-based metabolomics study was analyzed as described previously^22^. Briefly, 1 × 10^6^ HESC and 12Z cells were seeded and expanded on 6-well plates. After reaching 100% confluency, the cells were exposed to NAC, and IFN-ɣ for an hour (*n* = 3). Then, the media were discarded, and the cells were washed by PBS then the metabolites extracted using methanol: water (9:1, v/v) mixtures. The samples were centrifuged at 14000 g for 10 min at 4 °C. 0.4 mL aliquots were dried out in Eppendorf tubes. The residues were methoxyaminated and derivatized with MSTFA (N-Methyl-N-(trimethylsilyl) trifluoroacetamide with 1% TMCS (trimethylchlorosilane). After derivatization, the samples were transferred into GC–MS vials and analyzed using GC–MS (Shimadzu GCMS-QP2010 Ultra) with a DB-5MS stationary phase column (30 m + 10 m DuraGuard × 0.25 mm i.d. and 0.25-µm film thickness). Once the analysis was completed, complex chromatograms were deconvoluted, retention time corrected, and data matrix creation was performed using MS-DIAL software. The data matrices obtained from GC-MS were normalized to the total peak area and transferred to the SIMCA-P+ (v13.0, Umetrics, Umea, Sweden) program for multivariate analysis such as principal component analysis (PCA) and partial least squares differentiation analysis (PLS-DA) for the assessment of separability of the groups. The variable importance in projection (VIP) values was estimated to distinguish the most important metabolites for the stratifications of the groups, and regression coefficients were exploited to illustrate the effects of metabolites on the group. *Homo sapiens* associated pathway analyses were performed with significantly changed metabolites (*p* < 0.05) using the Human Metabolome Database^23–25^.

### Statistical analysis

We analyzed all data using the GraphPad Prism (v.10, GraphPad Software, San Diego, CA, USA). Normality of the data was checked by Shapiro-Wilk test. For normally distributed data, we performed one-way ANOVA to compare the differences between the groups, followed by Tukey’s post-hoc test for multiple comparisons. For non-normally distributed groups, the Kruskal-Wallis test was applied and Dunn’s post-hoc test was performed for pairwise differences. For metabolomics Student’s t-test was used for pairwise comparisons. A p value < 0.05 was considered statistically significant.

## Results

### NAC, IFN-ɣ, and IFN-ɣ+NAC decreases endometriotic cells’ proliferation

**Fig. 1:**
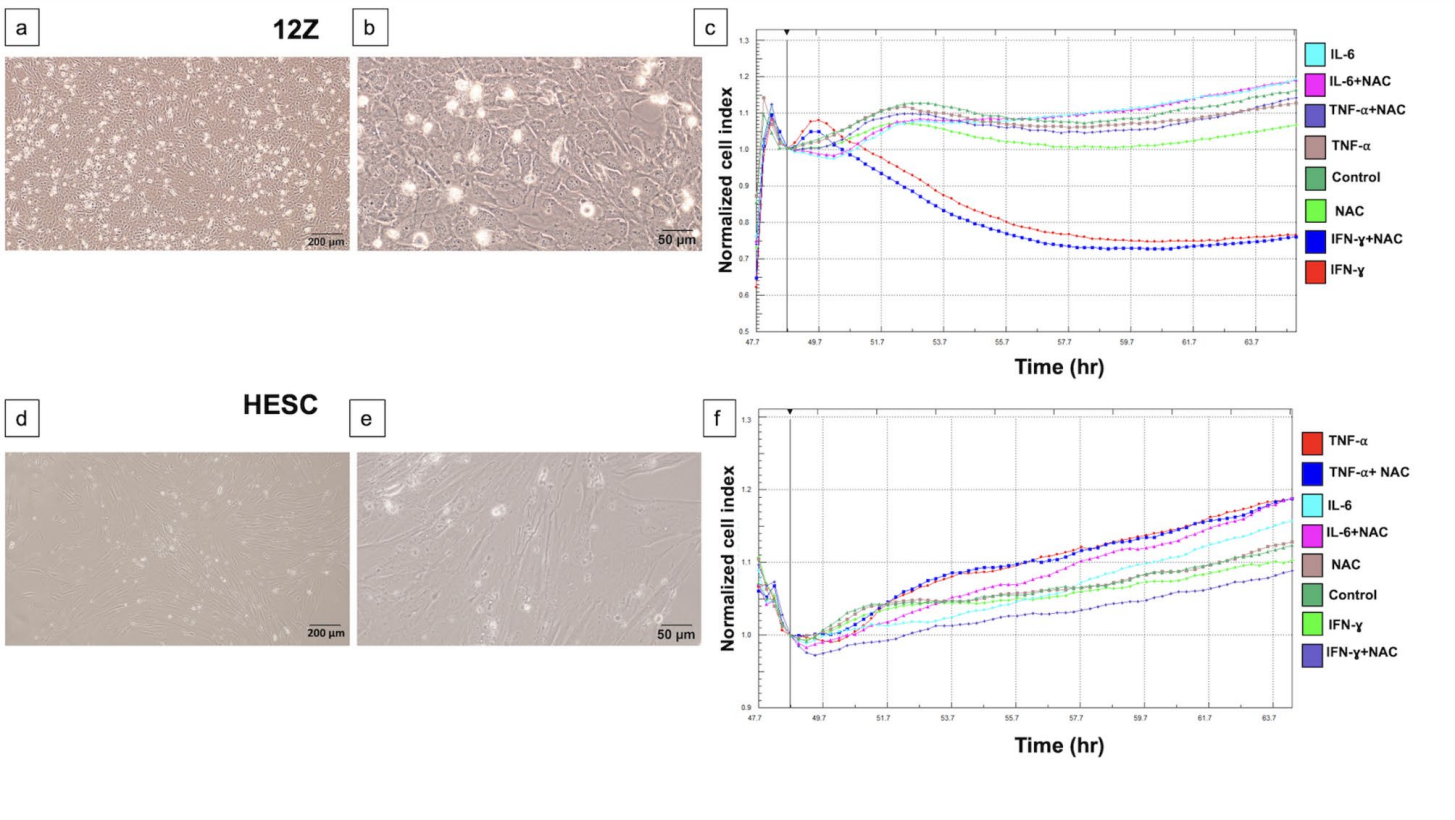
Phase-contrast microscopy images and proliferation analysis by xCELLigence of 12Z and HESC cells (**a**, **b**) 12Z cells exhibiting a smaller size, rounded nuclei, prominent nucleoli compared to (**d**, **e**) HESC cells. scale bars: 50 μm (100x) and 200 μm (400x). The xCELLigence system demonstrates the time- and dose-dependent antiproliferative efficacy of NAC and IFN-ɣ on endometriotic cells. (**c**) NAC reduced proliferation of 12Z cell compared to untreated cells. IFN-ɣ and the IFN-ɣ+NAC combination had an anti-proliferative effect on 12Z cells. Compared to the control group, cell proliferation was enhanced by IL-6 and IL-6 + NAC treatments. TNF-⍺ and TNF-⍺+NAC treatment resulted in a cell index like that of the untreated control group. (**f**) NAC treatment had no significant effect on HESC cell proliferation compared to the untreated control group. IFN-ɣ had a similar effect on cell proliferation as the untreated control. The combination of IFN-ɣ and NAC reduced the growth of endometriotic cells (*n* = 5).

We expanded the human endometriotic stromal cells (12Z) and human endometrial stromal cells and examined under a phase contrast microscope three times a week till they reached 70–80% confluency (Fig. 1a, b, d, e).

NAC reduced 12Z cell proliferation, as shown by real-time cell impedance. IFN-ɣ and the IFN-ɣ+NAC combination presented an antiproliferative effect on 12Z cells. IL-6 and IL-6 + NAC treatments increased cell proliferation compared to the control group. The cell index obtained by the application of TNF-⍺ and TNF-⍺+NAC was comparable to that of the untreated control group (Fig. 1c).

In HESC cells, NAC application did not alter the proliferation of the cells compared to the untreated control. The effect of IFN-ɣ on the proliferative capacity of the cells was comparable to that of the untreated control. IFN-ɣ+NAC combination decreased the proliferation of the endometriotic cells compared to the untreated control. IL-6, IL-6 + NAC, TNF-⍺ and TNF-⍺+NAC increased cellular proliferation compared to the control (Fig. 1f).

### NAC reduces IL-6- and TNF-⍺- induced apoptosis but enhances IFN-ɣ mediated apoptosis in endometriotic and endometrial stromal cells

**Fig. 2:**
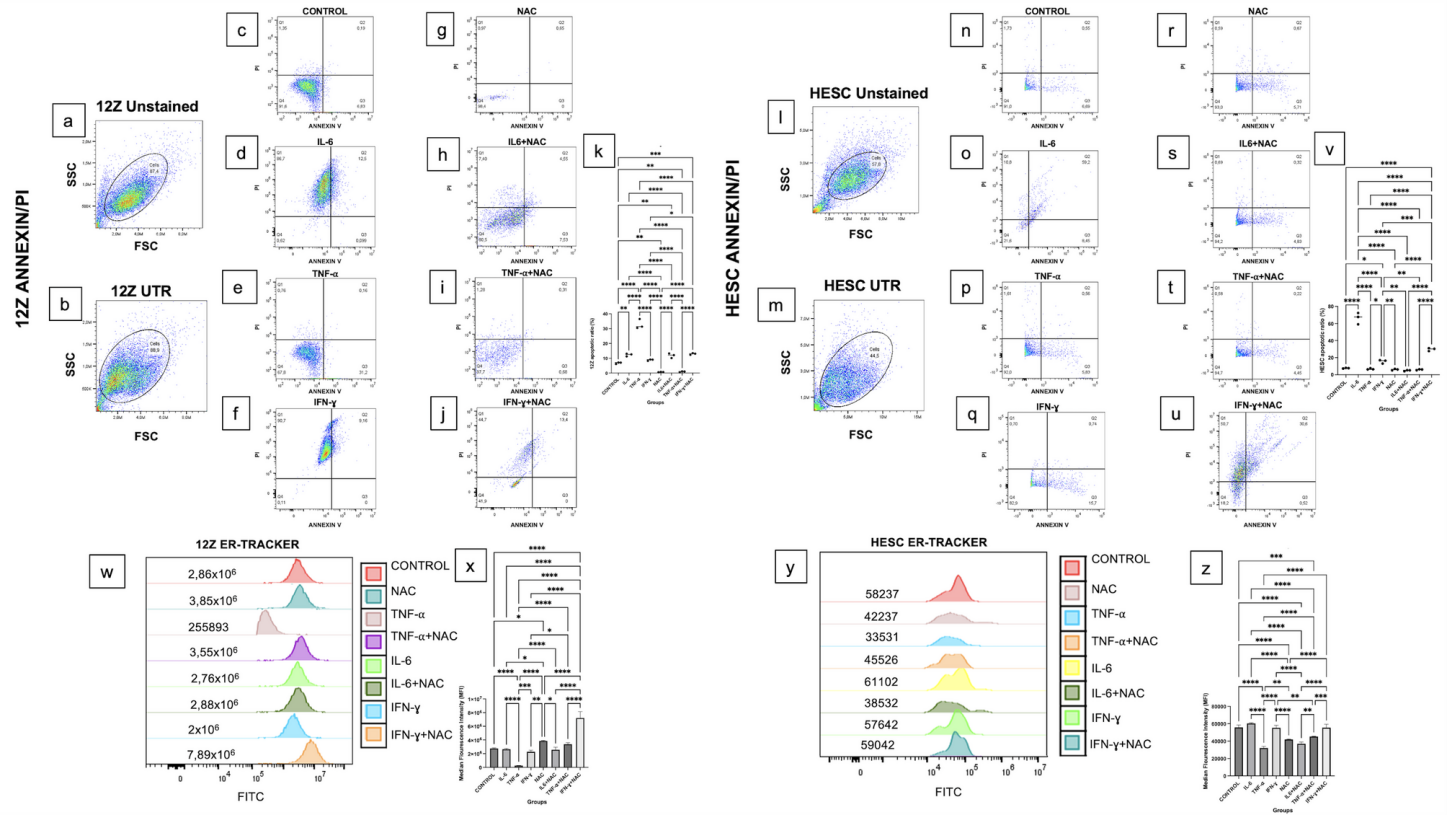
Annexin V/PI labeling and ER tracking reveal apoptosis and ER activity in 12Z and HESC cells under the influence of NAC and cytokines by flow cytometry (**a**, **b**) Unstained and untreated (UTR) 12Z cells, gated cell population selected regarding unstained cells (**c**) Annexin V/PI labeling of untreated 12Z cells. (**d–j**) 12Z cells treated with IL-6, TNF-⍺, IFN-ɣ, NAC, IL-6 + NAC, TNF-⍺+NAC, and IFN-ɣ+NAC showing varied apoptotic responses to treatments (**k**) Statistical comparison of total apoptotic ratios between groups. The control group exhibited lower apoptotic ratio than IL-6 and TNF-⍺, but higher than NAC. TNF-⍺ induced significantly higher apoptosis than all other treatments except IL-6 and IFN-ɣ+NAC. IFN-ɣ+NAC treatment resulted in the highest apoptotic ratio, while NAC alone showed the lowest. (**l**, **m**) Unstained and untreated HESC cells, Unstained and untreated (UTR) 12Z cells, gated cell population selected regarding unstained cells (**n**) Annexin V/PI labeling of untreated HESC cells. (**o–u**) HESC cells treated with IL-6, TNF-⍺, IFN-ɣ, NAC, IL-6 + NAC, TNF-⍺+NAC, and IFN-ɣ+NAC exhibiting distinct apoptotic responses (**v**) Statistical comparison of total apoptotic ratios between groups. The apoptotic ratio of the control group was lowest and highest in the IL-6 treated group significantly. TNF-⍺ induced less apoptosis than IFN-ɣ and IFN-ɣ+NAC. IFN-ɣ induces apoptosis was higher than that of NAC, IL-6 + NAC, and TNF-⍺+NAC but lower than IFN-ɣ+NAC. IFN-ɣ+NAC treatment induced significantly more apoptosis compared to the other treatment groups, except IL-6. (**w**, **x**) ER-tracker labeling in the NAC treated and IFN-ɣ +NAC treated group was higher than in the untreated 12z cells. (**y**, **z**) In HESC cells, alone or cotreatment of NAC via cytokines and IFN-ɣ did not alter the ER-tracking significantly. Percentages of total apoptotic cells assessed by flow cytometry after Annexin V/PI labeling. Data are shown as mean ± standard error of the mean (SEM). The normalized median fluorescence intensity (MFI) of the FITC-labeled cell population was evaluated. All statistical differences between groups are indicated by bars and are significant at **p < 0.05, **p ≤ 0.01 and ***p ≤ 0.001 levels (*n* = 3). Beckman Coulter Cytoflex Cytometer Software (v 1.2.5.3891) was used to create Flow Cytometry quadrants and histogram images, and GraphPad Prism (v 10.4.1) was used to create statistical graphics.

The Annexin-V apoptosis assay detected early and late apoptotic cells in the 12Z cell line following treatments with IL-6, TNF-⍺, IFN-ɣ (Fig. 2a–j). The groups were compared statistically by the ratios of total apoptotic cells’ percentages. The control group exhibited a significantly lower apoptotic ratio compared to the IL-6 and TNF-⍺ applied cells but higher than NAC applied cells (Fig. 2c, k). IL-6 induced apoptosis was significantly higher than TNF-⍺+NAC (Fig. 2d, k) (*p* < 0.05). TNF-⍺ induced apoptosis was significantly higher than IFN-ɣ, NAC, IL-6 + NAC, TNF-⍺+NAC, and IFN-ɣ+NAC (Fig. 2e, k) (*p* < 0.05). IFN-ɣ induced apoptosis was significantly higher than NAC and TNF-⍺+NAC but lower than IFN-ɣ+NAC (Fig. 2f, k) (*p* < 0.05). NAC alone had a significantly lower apoptotic ratio compared to IL-6 + NAC and IFN-ɣ+NAC (Fig. 2g, k) (*p* < 0.05). IL-6 + NAC induced significantly more apoptosis than TNF-⍺+NAC (Fig. 2h, k) (*p* < 0.05). IFN-ɣ+NAC exhibited the higher apoptotic ratio compared to those of the groups, including control, TNF-⍺, IFN-ɣ, NAC, and TNF-⍺+NAC (Fig. 2c, e–g, i–k) (*p* < 0.05).

In HESC cells (Fig. 2l–u), the control group exhibited a significant lower apoptotic ratio compared to the IL-6, IFN-ɣ, and IFN-ɣ+NAC (Fig. 2n, v) (*p* < 0.05). IL-6 induced apoptosis was significantly higher than TNF-⍺, IFN-ɣ, NAC, IL-6 + NAC, TNF-⍺+NAC and IFN-ɣ+NAC (Fig. 2o, v) (*p* < 0.05). TNF-⍺ induced apoptosis was significantly lower than IFN-ɣ, and IFN-ɣ+NAC (Fig. 2p, v) (*p* < 0.05). IFN-ɣ induced apoptosis was significantly higher than NAC, IL-6 + NAC, TNF-⍺+NAC but lower than IFN-ɣ+NAC (Fig. 2q, v) (*p* < 0.05). IFN-ɣ+NAC induced significantly more apoptosis compared to TNF-⍺, IFN-ɣ, NAC, IL-6 + NAC, TNF-⍺+NAC but lower than IL-6 (Fig. 2n, p-v) (*p* < 0.05).

### NAC and IFN-ɣ synergistically enhance ER activity in endometriotic cells

The cells were analyzed for ER stress using ER- tracking, and the normalized median fluorescence intensity (MFI) of the FITC-labeled cell population was evaluated. In 12Z cells, the control group (used as reference) exhibited significantly higher MFI than TNF-⍺ treated group, lower MFI than the NAC and IFN-ɣ+NAC groups. The IL-6 treatment showed significantly higher MFI compared to TNF-⍺, but lower than NAC and IFN-ɣ+NAC group. IFN-ɣ treatment demonstrated significantly higher ER-tracker labeling than TNF-⍺, but lower than NAC, TNF-⍺+NAC, and IFN-ɣ+NAC. NAC treatment showed significantly higher ER-tracker labeling than IL-6 + NAC, but lower than IFN-ɣ+NAC. IL-6 + NAC exhibited significantly lower MFI than IFN-ɣ+NAC and TNF-⍺+NAC exhibited significantly lower MFI than IFN-ɣ+NAC (Fig. 2w, x) (*p* < 0.05).

In HESC cells, TNF-⍺, NAC, IL-6 + NAC, and TNF-⍺+NAC reduced MFI compared to the control significantly. IL-6 treatment significantly elevated MFI, compared to the TNF-⍺, NAC, IL-6 + NAC, and TNF-⍺+NAC. TNF-⍺ reduced MFI compared to the IFN-ɣ, NAC, TNF-⍺+NAC, and IFN-ɣ+NAC. IFN-ɣ exhibited higher MFI compared to the NAC, TNF-⍺+NAC, and IL-6 + NAC significantly. NAC exhibited lower MFI compared IFN-ɣ+NAC significantly. IL-6 + NAC exhibited lower MFI compared to TNF-⍺+NAC, and IFN-ɣ+NAC significantly. TNF-⍺+NAC was significantly lower than IFN-ɣ+NAC (Fig. 2y, z) (*p* < 0.05).

### NAC and IFN-ɣ regulate proliferation and IRE1- mediated ER stress in endometriotic cells

**Fig. 3:**
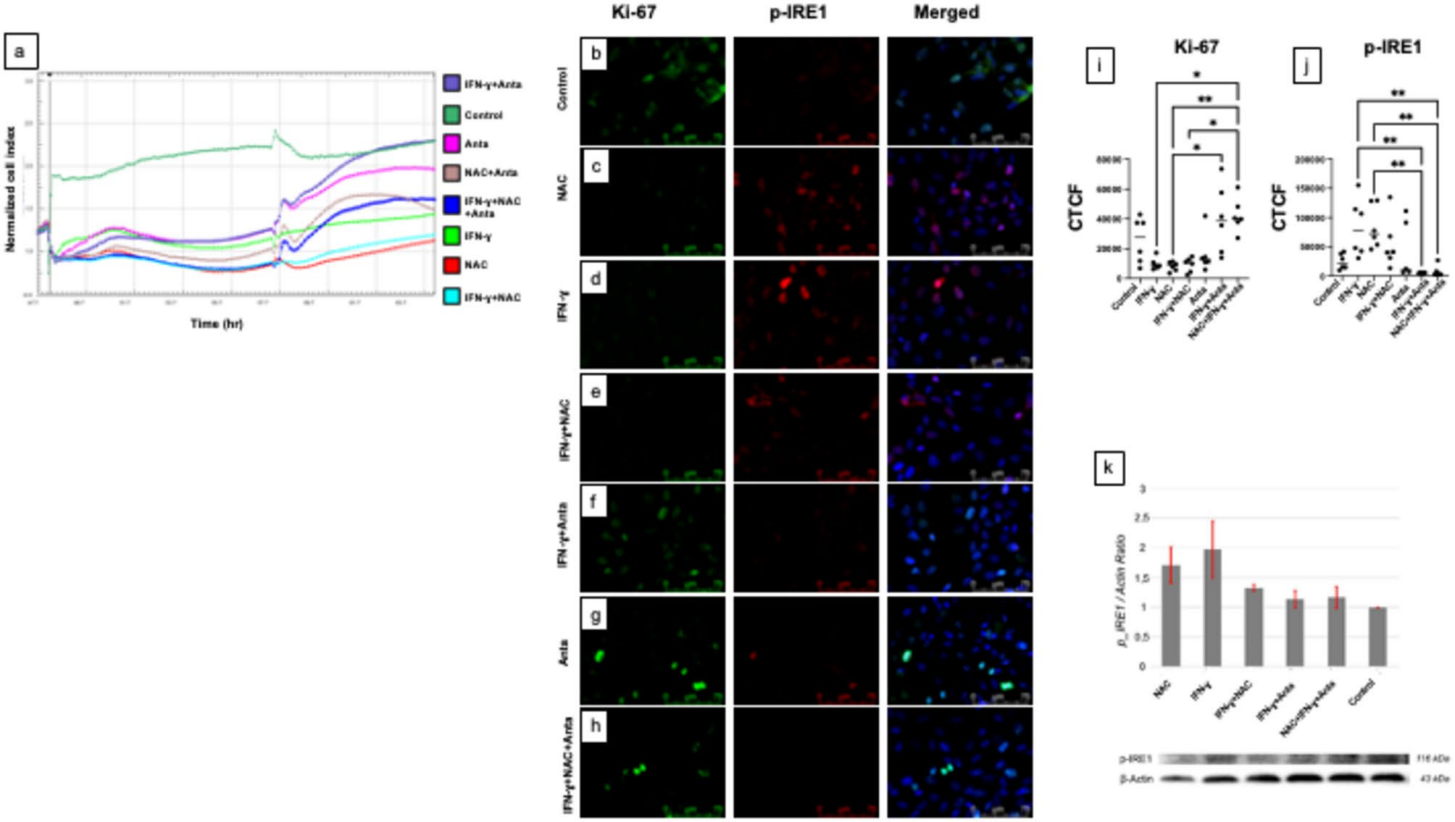
Effects of NAC, IFN-ɣ and IFN-ɣ receptor antagonist (Anta) on proliferation and IRE1 signaling in 12Z cells (**a**) xCELLigence analysis of 12Z cells individually and in combination with NAC, IFN-ɣ, Anta. Blocking IFN-ɣ signaling reduced IFN-ɣ and IFN-ɣ+NAC mediated antiproliferative effects (**b–j**) Ki-67 (green) and p-IRE1 (red) immunofluorescence labeling of 12Z cells. NAC, IFN-ɣ, and IFN-ɣ+NAC treatments decreased Ki-67 labeling compared to Anta including groups, significantly while p-IRE1 expression was significantly higher in IFN-ɣ-treated cells compared to the IFN-ɣ+Anta and IFN-ɣ+NAC + Anta groups. NAC treatment also increased p-IRE1 levels compared to the IFN-ɣ+Anta and IFN-ɣ+NAC + Anta groups. DAPI (blue) Scale bars: 75 μm (630x). (*n* = 3) Statistical differences between groups are indicated by bars and are significant at *p < 0.05, and **p ≤ 0.01 levels. (**k**) Representative western blotting analysis of p-IRE1 levels were relatively highest in IFN-ɣ treated cells, followed by NAC, IFN-ɣ+NAC, IFN-ɣ+NAC + Anta, IFN-ɣ+Anta, and the control groups. The bar graph shows that IRE1 protein level normalized to the β-actin protein (*n* = 3). Agilent RTCA Software (v 2.0) was used to create xCELLigence images, and GraphPad Prism (v 10.4.1) was used to create statistical graphics. ImageJ (v 1.54 g) was used to create immunofluorescence images and western blotting images.

In 12Z cells, blocking IFN-ɣ signaling by receptor antagonist (Anta) reduced antiproliferative impact of IFN-ɣ on endometriotic cells, and adding Anta to IFN-ɣ+NAC combination reduced antiproliferative efficacy of IFN-ɣ+NAC respectively (Fig. 3a).

Ki-67 immunofluorescence labeling was significantly lower in 12Z cells treated with NAC, IFN-ɣ, and the combination of IFN-ɣ+NAC compared to the IFN-ɣ+NAC + Anta group. NAC alone showed a significantly lower Ki-67 immunolabeling compared to the Anta group (Fig. 3b–i).

The p-IRE1 expression was significantly higher in IFN-ɣ treated cells compared to the IFN-ɣ+Anta and IFN-ɣ+ NAC + Anta groups. Also, NAC treatment increased p-IRE1 labeling, compared to the IFN-ɣ+Anta and IFN-ɣ+NAC + Anta groups (Fig. 3b–h, j).

The IFN-ɣ applied cells presented highest p-IRE1 levels by western blotting, followed by NAC, IFN-ɣ+NAC, IFN-ɣ+NAC + IFN-ɣ receptor antagonist, IFN-ɣ+ Anta and the control, relatively (Fig. 3k) (also see Supplementary Fig. 1).

### NAC and IFN-ɣ regulate metabolomics pathways and induce ER and mitochondrial stress in endometriotic cells

**Fig. 4:**
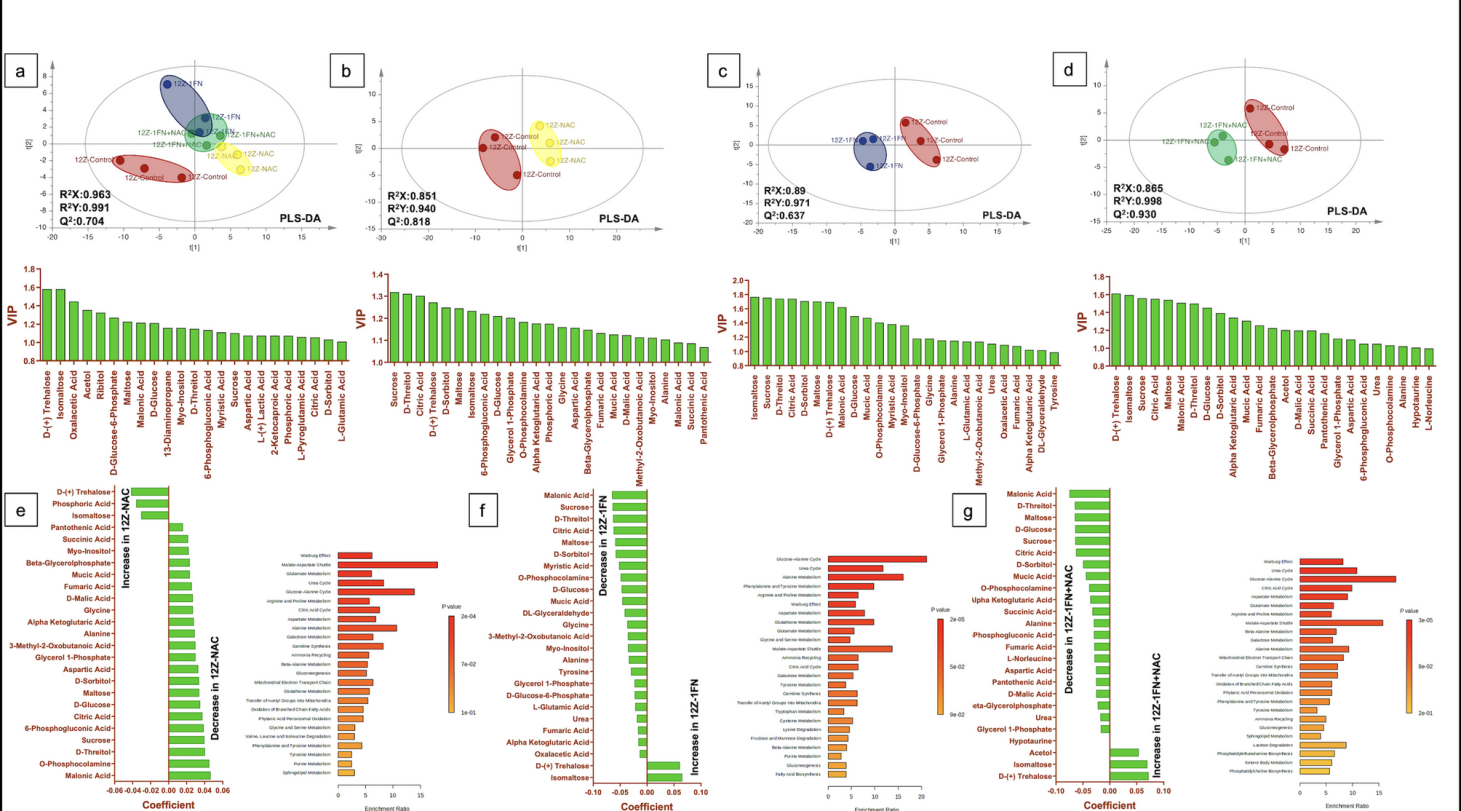
(**a**) Metabolomics analysis of 12Z cells revealed the altered metabolic processes (**b**, **e**) NAC treatment reduced key metabolic processes, including the Warburg effect, citric acid cycle, glutathione metabolism, and mitochondrial electron transport chain. (**c**, **f**) IFN-ɣ treatment decreased processes such as the urea cycle, glucose-alanine cycle, and malate-aspartate shuttle. (**d**, **g**) Combined IFN-ɣ+NAC treatment further suppressed pathways, including arginine-proline metabolism, branched-chain fatty acid oxidation, and phosphatidylethanolamine biosynthesis. (*n* = 3) (*p* < 0.05). The images were created via MetaboAnalyst (v 6.0).

Several metabolic pathways altered in 12Z cells when we applied NAC, IFN-ɣ and IFN-ɣ+NAC compared to the untreated control group (Fig. 4a). NAC, IFN-ɣ, and IFN-ɣ+NAC altered several common metabolic pathways in 12Z cells, including the Warburg effect, malate-aspartate shuttle, glutamate metabolism, urea cycle, glucose-alanine cycle, arginine-proline metabolism, citric acid cycle, alanine metabolism, galactose metabolism, carnitine synthesis, beta-alanine metabolism, and acetyl group transfer to mitochondria (Fig. 4b–g). Both NAC and IFN-ɣ reduced glutathione metabolism, aspartate metabolism, and ammonia recycling. NAC uniquely impacted pathways such as gluconeogenesis, mitochondrial electron transport chain, branched-chain fatty acid oxidation, phytanic acid peroxisomal oxidation, and glycine-serine metabolism. IFN-ɣ alone influenced tyrosine metabolism, glycine-serine metabolism, phenylalanine-tyrosine metabolism, tryptophan metabolism, cysteine metabolism, and lysine degradation. IFN-ɣ+NAC further altered lactose degradation and phosphatidylethanolamine biosynthesis.

## Discussion

Endometriosis is still an incurable and life-limiting health problem in women of reproductive age, yet its pathophysiology remains incompletely understood. Current treatment options are limited, underscoring that we need novel therapeutic approaches. To date, research has demonstrated several therapeutic benefits of NAC, including antioxidant activity, support of the activity of misfolded proteins and antimigratory, analgesic, anti-angiogenic, and anti-proliferative effects on endometriotic cells and tissues^19,20,26,27^. However, the mechanism behind the NAC’s anti-proliferative effects on endometriotic cells remains unclear currently. In this study, we investigated the effects of NAC and IFN-ɣ on endometriotic cells, particularly focusing on cell proliferation, apoptosis, endoplasmic reticulum (ER) stress, and metabolic alterations.

We performed xCELLigence proliferation analysis to evaluate the impact of NAC on the proliferative capacity of endometriotic cells in response to inflammatory cytokines in a time- and dose-dependent manner. We found that NAC reduced the cell index compared to the untreated group, consistent with our previous findings^20^. IFN-ɣ, both individually and in combination with NAC reduce endometriotic cell proliferation compared to the IL-6 and TNF-α groups. Notably, IL-6 enhanced the cell index of 12Z cells relative to the untreated group, aligning with previous research linking elevated IL-6 levels to increased proliferation of endometriotic cells in the late stages of the disease^28^. However, NAC did not reverse IL-6-induced proliferation, suggesting that NAC may not counteract IL-6 driven proliferation in endometriotic cells. Similarly, TNF-α, a cytokine known to promote endometriotic cell proliferation and ectopic localization^18^, resulted in a compatible cell index compared to the control. Although previous research suggests that NAC reduces peritoneal TNF-α levels^29^, NAC in combination with TNF-α did not decrease 12Z cells’ proliferation. This indicates that NAC may not sufficiently modulate TNF-α-driven proliferation in this context. IFN-ɣ, which is abundant in the early endometriotic microenvironment^30,31^, decreased the cell index within approximately 16 h in the present study. This effect was further enhanced by NAC, in consistence with previous findings that NAC exhibits antiproliferative properties on endometriotic cells and tissues^18–20^. However, the combined effect of NAC and IFN-ɣ on endometriotic cells has not been previously reported. Our findings introduce a novel perspective on the interaction between NAC and inflammatory cytokines in regulating endometriotic cell proliferation, highlighting the need for further investigation into IFN-ɣ signaling in endometriotic cells and its modulation by NAC. In HESC cells, NAC and IFN-ɣ did not individually affect the proliferation, unlike the response of 12Z cells to both compounds. However, their combination reduced the proliferation compared to the control. Conversely, IL-6, IL-6 + NAC, TNF-⍺, and TNF-⍺+NAC increased cell proliferation relative to the control.

The apoptotic analysis via flow cytometry revealed distinct responses among treatment groups, indicating that NAC modulates cytokine-induced apoptosis. IFN-ɣ+NAC significantly increased apoptotic ratios in both 12Z and HESC cells. In contrast, NAC alone exhibited a protective effect against IL-6- and TNF-α-induced apoptosis in both cell lines. In 12Z cells, IL-6 treatment significantly increased apoptosis compared to TNF-α + NAC. TNF-α-induced apoptosis was higher than IFN-ɣ, NAC, and other NAC-cytokine combination groups. These findings indicate that while IL-6 enhances apoptosis in 12Z cells, the presence of NAC weakens this effect. Interestingly TNF-α did not significantly alter apoptosis, suggesting that it may contribute to survival rather than apoptosis in endometriotic cells. In HESC cells, IFN-ɣ and IFN-ɣ+NAC significantly increased apoptosis compared to TNF-α and IL-6 + NAC. The IFN-ɣ+NAC combination exhibited the highest apoptotic ratio among all groups. Liu et al. reported that N-acetylcysteine (NAC) reversed tramadol-induced apoptosis in an endometrial cancer cell line previously^32^. Extending these findings, our study demonstrates that NAC modulates apoptosis in a cell-type and cytokine-specific manner. Specifically, NAC reduced IL-6-induced apoptosis in HESC cells but not in 12Z cells, and decreased TNF-α-induced apoptosis in 12Z cells but not in HESC cells.

To explore the underlying mechanism, we examined ER stress markers in NAC treated cells because our recent findings suggest that the reduced proliferation rate may not be due to pro-apoptotic effect but rather induce organelle stress. In our previous study, we observed a significant increase in GRP78 immunofluorescence labeling in 12Z cells following NAC treatment^20^, suggesting an association between NAC and organelle stress responses. In the present study, we observed a significantly increased ER-tracking signal in NAC and IFN-ɣ+NAC treated 12Z cells, supporting the hypothesis that NAC induces ER activity in a cytokine-dependent manner. Notably, IFN-ɣ alone elevated ER-tracker labeling, but its combination with NAC showed the highest ER-tracker signaling among all groups. IL-6 treatment moderately increased ER-tracker labeling compared to TNF-α but remained lower than NAC and IFN-ɣ+NAC-treatment. TNF-α treatment had the lowest ER-tracker labeling in 12Z cells, indicating a potential suppressive impact on ER activity. These results indicate that NAC-induced ER activity is potentiated by IFN-ɣ, but not by IL-6 or TNF-α. This highlights a specific interaction between NAC and IFN-ɣ in modulating ER stress responses. In HESC cells, NAC, TNF-α, IL-6 + NAC, TNF-α + NAC treatments significantly reduced ER-tracker labeling compared to the untreated group, pointing diminished ER response. However, IFN-ɣ exhibited a significantly higher ER-tracker signaling than NAC, TNF-α + NAC, and IL-6 + NAC. IFN-ɣ+NAC treatment led to the highest ER-tracker labeling in both 12Z and HESC cells, suggesting a synergistic impact on enhancing ER activity. These findings together suggest that NAC, particularly alone or combined with IFN-ɣ, enhances ER stress response in endometriotic cells.

To further explore antiproliferative effects of IFN-ɣ and IFN-ɣ+NAC on endometriotic cells we applied IFN-ɣ receptor antagonist (Anta). Our results showed that blocking IFN-ɣ receptor significantly reduced the antiproliferative effects of both IFN-ɣ and IFN-ɣ+NAC, analyzed by xCELLigence. Additionally, Ki-67 immunolabeling was significantly lower in the NAC, IFN-ɣ, and IFN-ɣ+NAC treated groups compared to IFN-ɣ+NAC + Anta applied cells.

As one of the transmembrane proteins of endoplasmic reticulum, IRE1-⍺ is triggered when the organelle is under stress^33^. To evaluate the ER stress activity, we performed p-IRE1-⍺ immunolabeling and western blotting on 12Z cells. The p-IRE1-⍺ immunofluorescence labeling was higher in the IFN-ɣ and NAC treated cells compared to other groups and this effect was antagonized by Anta. These findings were consistent with Saha et al., who reported that oral NAC (50–100 mg/kg) with high fat diet increased IRE1-⍺ immunofluorescence labeling and protein expression by western blotting^33^. Our results aligned with those results, suggesting that NAC and IFN-ɣ can drive endometriotic cells to endoplasmic reticulum stress in a dose-dependent manner. Western blotting further confirmed that p-IRE1-⍺ levels were highest in the IFN-ɣ treated cells, followed by NAC, and IFN-ɣ+NAC treatments, with levels notably reduced in the Anta applied groups and the control. These findings highlight that IFN-ɣ mediated and IFN-ɣ+NAC induced ER stress is dependent on presence of IFN-ɣ.

Metabolomic data analyses revealed significant alterations in various biological processes within 12Z cells following treatments with NAC, IFN-ɣ, and IFN-ɣ+NAC. Notably, these treatments decreased several metabolic processes associated with energy metabolism, including malate-aspartate shuttle, citric acid cycle, amino acid metabolism, and mitochondrial function. The disruption in mitochondrial activity, such as mitochondrial transfer from stromal cells to tumor cells, is crucial to tumor cell survival^34^, and the malate-aspartate shuttle constitutes a significant source of ROS^35^. Cheng et al.^36^ suggested that NAC’s antiproliferative effect may be related to methylation of sulfhydryl groups rather than antioxidant or radical scavenging mechanism in cancer cells. The reduced mitochondrial activity and metabolic alterations observed in this study could be explained by thiol-dependent ROS signaling mechanisms similar to those seen in tumor cells^37^. This study revealed a decrease in nitrogen metabolism, glycine and serine metabolism, gluconeogenesis, the oxidation of branched-chain fatty acids, and phytanic acid peroxisomal oxidation, phenylalanine-tyrosine metabolism, and tyrosine metabolism, indicating mitochondrial damage. Zalewska et al. reported that NAC administration did not reverse the previously induced mitochondrial stress^38^, which is consistent with our findings.

The results revealed that the ER and mitochondrial function were significantly impacted by the administration of NAC in endometriotic cells. Specifically, significant enrichment observed in pathways associated with energy metabolism, including tricarboxylic acid (TCA) cycle intermediates and fatty acid β-oxidation and alterations in amino acid metabolism pathways, particularly those involving glutathione metabolism, emphasized the role of NAC in maintaining cellular redox homeostasis. Additionally, disruptions in lipid metabolic pathways, including phospholipid and sphingolipid metabolism, suggested a functional link to ER stress. These findings underscore the dual role of NAC in modulating mitochondrial and ER functions through its impact on critical metabolic pathways, providing a mechanistic basis for its therapeutic potential in endometriosis.

Although, NAC has been accepted as a promising immunotherapeutic agent^39^, its ability to activate PI3K/Akt and reduce ROS enhance the anti-tumor function of immune cells^40^. Our metabolomics results, that reveal alterations in the endometriotic cells’ amino acid and starvation pathways, organelle stress and cytokine-dependent efficacy of NAC, align with studies suggest its immunostimulatory effects are mediated through PI3K/Akt and ROS reduction. The dual roles that are immunostimulatory effect at low concentrations and a suppressive effect at higher concentrations may explain its co-efficacy with IFN-ɣ in endometriotic cells^36^. To fully understand NAC’s redox mechanisms, more advanced analytical techniques are needed.

Although our metabolomics data provide insights into the metabolic shifts induced by NAC and IFN-γ, it is important to acknowledge that 12Z and T-HESC cells are immortalized, similar to malignant cells. This may have influenced the metabolic alterations observed in our study. Therefore, these findings should be interpreted with caution when extrapolating to primary endometriotic lesions.

In conclusion, the current study is the first to interpret the cytokine-NAC interaction on endometriotic cells, highlighting the need for further research in this area. Our study provides insights into the interplay between NAC, IFN-ɣ, and endometriotic cells. NAC, alone or in combination with IFN-ɣ, exerted antiproliferative effects on endometriotic cells and induced ER stress and mitochondrial dysfunction. These effects were associated with alterations in cellular metabolism, including energy and amino acid metabolism, and redox balance. The potential therapeutic utility of NAC in treating endometriosis by targeting the inflammatory microenvironment and cellular metabolism is highlighted by our findings. However, further research is necessary to elucidate the underlying molecular mechanisms and validate the efficacy of NAC in clinical settings. Future studies should investigate the effects of NAC on other cell types and IFN-ɣ signaling pathways involved in the pathogenesis of endometriosis.

## Electronic supplementary material

Below is the link to the electronic supplementary material.

Supplementary Material 1

## Data Availability

The data presented in this study are available on request from corresponding author.
