# Supplementary figures and images for: N-acetylcysteine stimulates organelle malfunction in endometriotic cells via IFN-gamma signaling
Source: Sci Rep. 2025 Apr 29;15:15120. doi: 10.1038/s41598-025-00195-z (PMC12041191; doi:10.1038/s41598-025-00195-z)

**
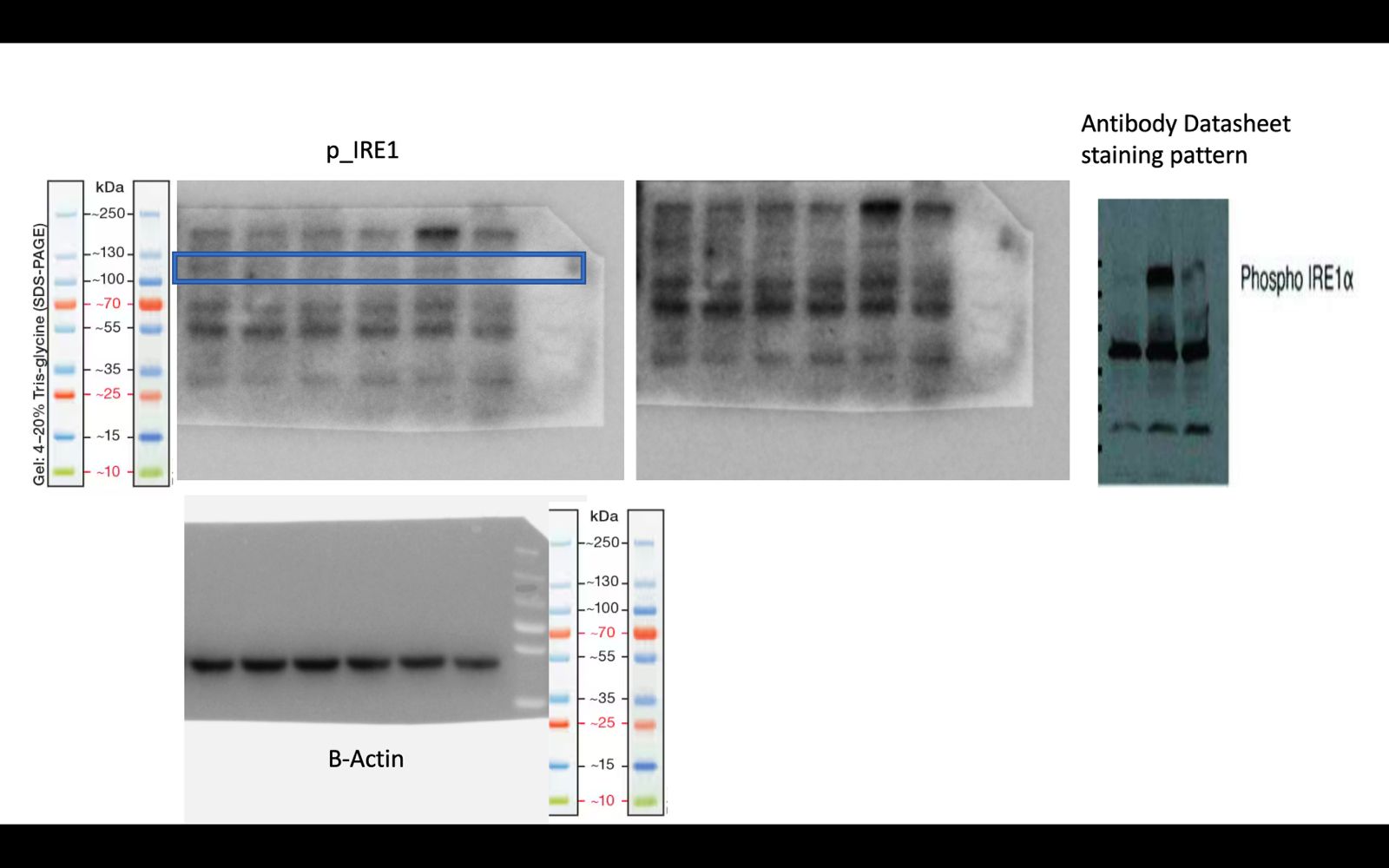
**

**Supplementary Fig. 1:** Supplementary information of western blotting analysis

Supplement: Supplementary file 1 — Supplementary Material 1 [file 41598_2025_195_MOESM1_ESM.docx]
